# Quality of Life and Participation of Children With Visual Impairment: Comparison With Population Reference Scores

**DOI:** 10.1167/iovs.62.7.14

**Published:** 2021-06-11

**Authors:** Ellen B. M. Elsman, Mitchel Koel, Ruth M. A. van Nispen, Ger H. M. B. van Rens

**Affiliations:** 1 Department of Ophthalmology, Amsterdam UMC, Vrije Universiteit Amsterdam, the Amsterdam Public Health Research Institute, MB Amsterdam, The Netherlands

**Keywords:** visual impairment (VI), children, quality of life, participation, Child and Adolescent Scale of Participation (CASP), KIDSCREEN-27 questionnaire

## Abstract

**Purpose:**

The purpose of this study was to investigate quality of life and participation in children aged 3 to 17 years with visual impairment (VI) compared to reference groups and between subgroups with increasing severity levels of VI.

**Methods:**

Parents of children aged 3 to 17 years (*n* = 500) and children aged 13 to 17 years (*n* = 75) completed the Child and Adolescent Scale of Participation (CASP). Children aged 7 to 17 years (*n* = 263) and their parents (*n* = 255) completed the KIDSCREEN-27 questionnaire to assess quality of life. Scores were compared to age and/or gender-appropriate population-based samples. For the CASP, a comparison was also made with children with chronic conditions or disabilities. The association between severity of VI and quality of life or participation was analyzed with linear regression models.

**Results:**

Children reported significantly worse on Physical Wellbeing and Social Support & Peers, but better on the School Environment KIDSCREEN-27 subscales compared to reference groups. Parents additionally reported worse on Autonomy & Parent Relation. Children's participation was significantly worse compared to a population-based sample, but significantly better compared to children with chronic conditions and disabilities. Having moderate or severe VI/blindness was significantly associated with worse participation, as reported by parents relative to those with no VI.

**Conclusions:**

Quality of life of children with VI is affected especially regarding Physical Wellbeing and Social Support & Peers compared to a reference population, and their participation is considerably worse. Participation was more affected in children with more severe VI. These results contribute to the understanding of the impact of VI. Interventions targeting physical health, social skills, and participation are warranted.

Although the prevalence of childhood visual impairment (VI) is low compared to older adults,^1^ it has lifelong implications. Several qualitative studies have investigated the impact of VI on particular life aspects of children with VI^2^^–^^6^ or their life as a whole.^7^^,^^8^ For example, Rainey et al. found that VI affects sensorial development, and physical, psychological, and social wellbeing, with variations in relevance of themes across different age groups.^8^

In recent years, the patient-based assessment of the impact of a condition on functioning, participation, and quality of life has become more important.^9^^,^^10^ Quality of life is a multidimensional construct consisting of physical, emotional, and social wellbeing.^11^^–^^13^ The International Classification of Functioning, Disability, and Health for Children and Youth (ICF-CY) has made the concept of participation relevant for children. The ICF-CY defines participation as “a person's involvement in life situations,” by performing activities which are defined as “the execution of tasks.”^13^^,^^14^ Quality of life and participation differ from each other, in that the former is more related to subjective experiences, and the latter to more objective tasks that can or cannot be performed. Both are important outcomes to assess the burden of a condition or the effectiveness of an intervention.^13^

To evaluate quality of life and participation in children with VI, both generic and disease-specific instruments can be used. Several vision-specific instruments for children with VI have been developed in recent years.^15^^–^^17^ These instruments are valuable for assessing vision-related problems and are probably more sensitive to the specific problems these children have. However, in order to compare results to other populations, generic instruments are more useful.

Despite the large number of qualitative studies conducted to investigate the impact of VI on particular life aspects, few quantitative studies have focused on the quality of life of children.^18^^–^^23^ These studies used relatively small sample sizes, some of them focused on particular eye conditions, and not all of them compared scores to a reference in the general population or a control group. Moreover, these studies focused on quality of life, whereas limitations in activities and participation are often used to assign children to pedagogical, behavioral, or low vision interventions. As such, limited conclusions can be drawn on whether quality of life and participation is different in children with VI compared to children in the general population.

Therefore, this study aims to evaluate quality of life and participation of children with VI aged 3 to 17 years and to compare them with relevant reference groups found in literature. Second, the associations between the severity of vision loss and quality of life and participation are investigated.

## Methods

Data for this study were collected as part of a larger study, aimed at validating the Participation and Activity Inventory for Children and Youth (PAI-CY) 3 to 6 years, 7 to 12 years, and 13 to 17 years.^15^^,^^24^^,^^25^ The instruments included in the present study were originally selected as comparator instruments for the PAI-CY. The Medical Ethical Committee of Amsterdam UMC, the Netherlands, approved the study protocol. The study adhered to the tenets of the Declaration of Helsinki. Written informed consent was received from all parents of children, and from 13 years onward also from the children themselves.

### Participants

Parents of children aged 3 to 17 years who were registered at Dutch low vision services (Royal Dutch Visio and Bartiméus) at the time of the study (2015–2017), were invited to participate. Referral to Dutch low vision services is based on the following national guidelines: having best corrected visual acuity <0.3, visual field <30 degrees, in case of disorders in lower or higher visual functions (e.g. respectively, night blindness/photophobia or cerebral VI), or in case of a progressive disorder, or in case of a rehabilitation need for which no opportunities in regular ophthalmological care exist.^26^ Participants had to have adequate knowledge and understanding of the Dutch language. Children with VI from any cause were eligible; no restrictions were applied regarding visual performance. Children with profound cognitive impairment, registered in patient files at the low vision services, were excluded from participation. Children with mild cognitive impairment, if reported by their parents but not registered in the patient's file, could participate.

### Procedures

Participating parents completed questionnaires through a web-based survey (a paper-and-pencil version was available on request), whereas children completed questionnaires through face-to-face interviews in their homes. The Dutch version of the Child and Adolescent Scale of Participation (CASP)^27^ was completed by parents of children aged 3 to 17 years and by children aged 13 to 17 years. The KIDSCREEN-27 questionnaire^28^ was completed by the parents of the children aged 7 to 17 years and the children themselves. Parents also completed questions regarding sociodemographic and clinical characteristics of their child. Ophthalmic diagnoses, decimal visual acuity, and visual field of the children were retrieved from the patient records at the low vision service. Missing data were complemented by self-reported data from parents (*n* = 33). Decimal visual acuity was classified in five levels based on the better-seeing eye, according to criteria of the World Health Organization (WHO): logMAR ≤0.3 referred to “no VI,” logMAR 0.31 to 0.52 to “mild VI,” logMAR 0.53 to 1 to “moderate VI,” logMAR 1.01 to 1.30 to “severe VI,” and logMAR ≥1.31 to “blind.”^29^

### Instruments

The KIDSCREEN-27 questionnaire was used to evaluate quality of life. The KIDSCREEN-27 contains five subscales: Physical Wellbeing (5 items); Psychological Wellbeing (7 items); Autonomy & Parent Relation (7 items); Social Support & Peers (4 items), and School Environment (4 items). The 27 items are rated on a five-point scale based on frequency or degree of feeling. Scores on the subscales are expressed as T-scores, with a mean of 50 and a standard deviation of 10. Higher T-scores indicate better quality of life.^30^

The CASP measures the degree of participation of a child in home, school, and community activities and asks respondents to compare their participation to the degree of participation of children of the same age. The CASP was originally developed to assess participation in children with acquired brain injury, but has been used in children with other conditions as well.^31^ A systematic review suggests that, at the time of this study, it is the most appropriate instrument to assess participation in children with disability.^32^ The CASP consists of 20 items rated on a four-point scale with response options “age expected,” “somewhat limited,” “very limited,” or “unable.” The response option “not applicable” is treated as a missing value. Higher scores reflect greater age-expected participation.^33^

### Statistical Analyses

Descriptive statistics were used to report sociodemographic and clinical characteristics of participants. Quality of questionnaire data was checked by assessing acquiescence bias (i.e. the tendency to opt for the same answer regardless of the content of an item).^34^ However, no indications for acquiescence bias were found, as variability in responses remained and the number of missing responses did not increase. Scores of participants on (sub)scales of the KIDSCREEN-27 and CASP were compared to reference scores found in literature using one-sample *t*-tests. For the KIDSCREEN-27, participants’ scores on subscales were compared to the Dutch reference population (*n* = 1813–1862, depending on subscale) in our primary analyses and age-range (i.e. 7–11 years and 12–17 years) and gender subpopulation reference scores to make the most direct comparisons in our secondary analyses.^30^ As the secondary analyses had a more explorative character, a correction for multiple testing was applied within each subscale using a Bonferroni correction (0.05/4 age- and gender subpopulations = 0.0125). Contrasting findings have been reported regarding the underlying factor structure for the CASP, with studies reporting a unidimensional scale, and three or four subscales.^31^^,^^33^^,^^35^ Therefore, the number of factors was assessed by performing an eigenvalue decomposition on the matrix of robust (Spearman) correlations between the items completed by parents. The acceleration factor along the scree plot was calculated,^36^ suggesting a one-factor solution. Subsequently, principal component analyses were performed to proxy if all items load on a single component. Principal components of the one-factor solution were all positive and high (>0.6), accounting for 66% of the explained variance. Two, three, and four-component solutions were forced upon the data but did not give reasons to select either of these options. Therefore, it was concluded that the 20 items reflected a unidimensional scale, and total scores of the CASP were calculated. Because of missing data due to the response option “not applicable,” sum scores were calculated when ≥75% of the items were completed. Respondents with <75% of the items completed were omitted in the analyses involving sum scores. No reference scores for the Dutch general population are available for the CASP. Therefore, scores of participants were compared to scores originating from two sources. First, scores of participants aged 3 to 11 years were compared to reference scores from a German population-based sample with the same age (*n* = 215).^35^ Second, scores of participants aged 12 to 17 years were compared to reference scores from a Canadian sample aged 11 to 17 years with different chronic conditions and disabilities (e.g. cerebral palsy, acquired brain injury, and autism spectrum disorder, *n* = 409).^37^ Self-report scores were compared to youth-report reference scores, whereas proxy-report scores were compared to parent-report reference scores. Clinical significance of the differences was investigated using Cohen's D. Effect sizes 0.2 to 0.49 were considered small, 0.5 to 0.79 were considered moderate, and ≥0.8 were considered large.^38^

The association between severity of VI (no VI; mild VI; moderate VI; and severe VI/blindness) on quality of life and participation was assessed using linear regression analysis. After checking relevant assumptions, the following independent variables were included in the corrected model: age (3–6 years, 7–12 years, and 13–17 years), gender (male or female), level of education of the parent (low, middle, or high) and comorbidity of any type (yes or no).

## Results

**Figure 1.**
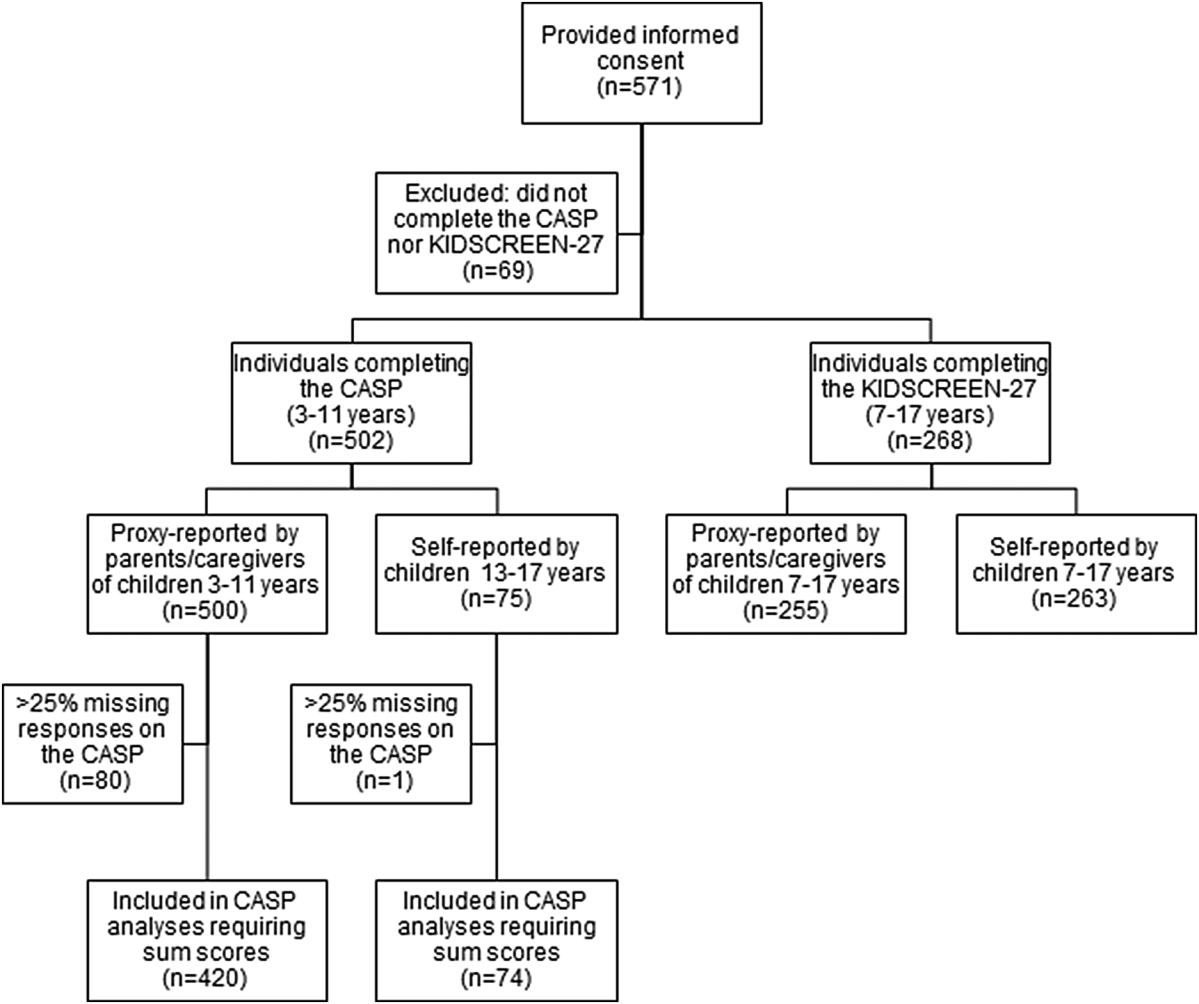
Flow chart of the participants.

Of the 571 consenting participants, 502 parents and/or children completed the CASP. Because of missing data due to the “not applicable” response option, sum scores could be calculated for 420 proxy-reports and 74 self-reports. The KIDSCREEN-27 was completed by 268 parents and/or children. Figure 1 presents the flow chart. Table 1 shows the sociodemographic and clinical characteristics of the study sample.

**Table 1.** Sociodemographic and Clinical Characteristics of Participants CASP (*n* = 502) and KIDSCREEN-27 (*n* = 268)

Participant Characteristics	CASP	KIDSCREEN-27
Age in years, mean ± SD (range)	7.81 ± 3.87 (3–17)	10.89 ± 2.82 (7–17)
Male gender, *n* (%)	293 (58.4)	159 (59.3)
Parent who completed questionnaire,^†^ *n* (%)		
Mother	385 (77.0)	190 (73.6)
Father	58 (11.6)	34 (13.2)
Together	46 (9.2)	27 (10.5)
Caregiver	11 (2.2)	7 (2.7)
Category of VI,^*^ *n* (%)		
Blindness: LogMAR ≥1.31	30 (6.0)	23 (8.6)
Severe VI: logMAR 1.01–1.30	14 (2.8)	4 (1.5)
Moderate VI: LogMAR 0.53–1	147 (29.3)	73 (27.2)
Mild VI: logMAR 0.31–0.52	99 (19.7)	50 (18.7)
No VI: logMAR ≤0.30	191 (38.0)	111 (41.4)
Unknown	21 (4.2)	7 (2.6)
Nationality,^†^ n (%)		
Dutch	473 (94.2)	241 (89.9)
Other	29 (5.8)	27 (10.1)
Financial situation,^†^ *n* (%)		
Usually enough money	266 (53.0)	122 (45.5)
Just enough money	107 (21.3)	63 (23.5)
Not enough money	26 (5.2)	19 (7.1)
I'd rather not tell	103 (20.5)	54 (20.1)
Comorbidity,^†^ *n* (%)	221 (44.0)	114 (42.5)
Cognitive impairment,^†^ *n* (%)	78 (15.5)	24 (9.0)
Primary cause of VI, *n* (%)		
Retina	147 (29.3)	91 (34.0)
Cerebral visual impairment (CVI)	87 (17.3)	44 (16.4)
Nystagmus	71 (14.1)	35 (13.1)
Lens	39 (7.8)	17 (6.3)
Optic nerve	33 (6.6)	20 (7.5)
Refraction	26 (5.2)	14 (5.2)
Strabismus	21 (4.2)	8 (3.0)
Glaucoma	7 (1.4)	4 (1.5)
Other	14 (2.8)	10 (3.7)
Unknown	57 (11.4)	25 (9.3)

* Visual impairment categories were mainly based on acuity loss in the better-seeing eye following the World Health Organisation.^29^

† Data is proxy-reported.

Table 2 shows the comparison of mean scores on the KIDSCREEN-27 with reference data. Children reported significantly worse scores compared to reference data for Physical Wellbeing and Social Support & Peers, but significantly better scores for School Environment. Effect sizes for these differences ranged from 0.18 to 0.36. Parents reported significantly worse scores compared to reference data for Physical Wellbeing, Autonomy & Parent Relation, and Social Support & Peers, but also significantly better scores for School Environment. Effect sizes for these differences ranged from 0.13 to 0.35.

**Table 2.** Comparison of Participants’ KIDSCREEN-27 Subscale Scores With Reference Data for the Total Population and Age-Range and Gender Subpopulations

		Physical Wellbeing				Psychological Wellbeing				Autonomy & Parent Relation				Social Support & Peers				School Environment			
Group	Number of Participants	Popul-ation Score, Mean (SD)	Refere-nce Score, Mean (SD)	*P* Value	Effect Size	Popul-ation Score, Mean (SD)	Refer-ence Score, Mean (SD)	*P* Value	Effect Size	Popul-ation Score, Mean (SD)	Refere-nce Score, Mean (SD)	*P* Value	Effect Size	Popul-ation Score, Mean (SD)	Refere-nce Score, Mean (SD)	*P* Value	Effect Size	Popul-ation Score, Mean (SD)	Refere-nce Score, Mean (SD)	*P* Value	Effect Size
Self-reported data																					
Total	263	51.2 (9.6)	52.9 (10.0)	**0.004**	0.18	53.0 (9.4)	52.8 (9.5)	0.711	−0.02	54.7 (10.4)	54.0 (9.5)	0.266	−0.07	50.1 (11.3)	52.4 (9.0)	**<0.001**	0.23	56.6 (10.2)	53.1 (9.7)	**<0.001**	−0.36
Girls 7–11	80	51.2 (8.4)	57.8 (9.5)	**<0.001**	**0.73**	52.3 (8.4)	55.3 (10.1)	**0.002**	0.32	53.6 (9.1)	55.9 (10.1)	0.023	0.25	50.3(10.5)	53.9 (8.6)	**0.001**	0.38	57.4 (9.3)	58.4 (9.4)	0.301	0.10
Boys 7–11	108	52.3 (9.5)	56.9 (9.6)	**<0.001**	0.48	52.6 (10.3)	54.9 (9.7)	0.025	0.23	53.6 (11.9)	54.7 (10.2)	0.380	0.09	49.7 (13.2)	52.6 (9.2)	0.023	0.26	57.2 (11.3)	56.8 (10.0)	0.730	−0.04
Girls 12–17	27	47.2 (10.6)	48.5 (8.9)	0.548	0.13	53.7 (8.8)	49.9 (8.4)	0.035	−0.44	58.8 (10.5)	52.7 (8.8)	**0.010**	−**0.58**	51.8 (6.07)	52.3 (8.9)	0.666	0.07	53.4 (10.0)	50.3 (8.5)	0.107	−0.35
Boys 12–17	48	50.8 (10.4)	52.5 (9.5)	0.256	0.17	54.7 (9.0)	53.3 (9.2)	0.270	−0.16	56.7 (8.0)	53.8 (9.3)	0.014	−0.34	49.5 (10.6)	51.3 (9.3)	0.235	0.18	55.8 (8.7)	50.8 (8.9)	**<0.001**	−**0.57**
Proxy-reported data																					
Total	255	49.4 (11.7)	53.2 (10.1)	**<0.001**	0.35	50.1 (11.6)	51.2 (10.0)	0.130	0.10	52.3 (9.7)	53.9 (9.3)	**0.006**	0.18	52.0 (10.3)	53.2 (8.8)	**0.048**	0.13	54.5 (10.8)	53.1 (9.9)	**0.036**	−0.14
Girls 7–11	77	51.0 (11.0)	56.5 (9.3)	**<0.001**	**0.54**	51.3 (11.4)	52.8 (9.7)	0.242	0.15	52.6 (9.4)	55.4 (9.4)	**0.011**	0.30	52.6 (9.4)	54.9 (7.9)	0.036	0.26	54.4 (10.4)	57.7 (9.4)	**0.007**	0.34
Boys 7–11	104	49.9 (11.6)	56.8 (8.6)	**<0.001**	**0.67**	48.5 (10.7)	52.5 (9.8)	**<0.001**	0.38	51.4 (9.1)	53.7 (9.3)	**0.009**	0.26	51.5 (10.4)	54.1 (7.9)	0.015	0.27	53.2 (11.2)	56.1 (9.9)	**0.010**	0.27
Girls 12–17	27	44.4 (10.3)	49.0 (10.1)	0.030	0.45	50.3 (11.3)	49.6 (9.9)	0.728	−0.07	51.5 (6.6)	53.0 (9.1)	0.228	0.20	50.1 (10.4)	52.7 (8.5)	0.197	0.28	58.4 (10.3)	50.8 (9.6)	**0.001**	−**0.76**
Boys 12–17	46	47.8 (12.8)	53.7 (9.7)	**0.003**	**0.52**	51.2 (13.4)	51.4 (10.0)	0.897	0.02	53.6 (12.3)	54.2 (9.3)	0.738	0.06	52.9 (11.5)	52.3 (9.8)	0.751	−0.05	55.2 (10.6)	51.3 (9.3)	0.014	−0.40

Bold is significant at *P* < 0.05 for the total group and *P* < 0.013 for subgroups (Bonferroni correction) and/or represents a moderate/large effect size (Cohen's D ≥0.5).

When looking at the age-range and gender subpopulation scores, boys and girls aged 7 to 11 years scored worse on almost all subscales compared to the reference data, for both the self-report and proxy-report data. Significantly worse scores for both boys and girls in this age-category were found for Physical Wellbeing self-report and proxy-report, Autonomy & Parent Relation proxy-report, and School Environment proxy-report. Effect sizes for these differences ranged from 0.26 to 0.73. For the age group 12 to 17 years, differences were smaller. Girls self-reported significantly better scores for Autonomy & Parent Relation, whereas boys reported significantly better scores for School Environment. Effect sizes for these differences were, respectively, 0.58 and 0.57. Parents of children aged 12 to 17 years reported significantly worse scores for Physical Wellbeing for boys and significantly better scores for School Environment for girls. Effect sizes for these differences were, respectively, 0.52 and 0.76.

Table 3 presents the comparison of mean scores on the CASP with reference data. Parents of children aged 3 to 11 years reported significantly worse scores compared to a reference population. The effect size was large. Parents of children aged 12 to 17 years reported significantly better scores compared to a reference population with different chronic conditions and disabilities. The effect size was large. Similarly, children aged 13 to 17 years also reported significantly better scores compared to this reference population. The effect size was large.

**Table 3.** Comparison of Participants’ CASP Scores With Reference Data

Group	Number of Participants	Population Score, Mean (SD)	Reference Score, Mean (SD)	*P* Value	Effect Size
Proxy-reported 3–11 y	329	80.3 (23.3)	98.2 (5.8)^*^	<0.001	1.58
Proxy-reported 12–17 y	91	83.2 (16.3)	63.5 (12.8)**^†^**	<0.001	−1.90
Self-reported 13–17 y	74	89.6 (10.2.1)	69.5 (8.2)**^†^**	<0.001	−3.06

* Compared to a population-based sample of German children aged 3–11 years.^35^

† Compared to a sample of Canadian children aged 11–17 years with different chronic conditions or disabilities.^37^

**Figure 2.**
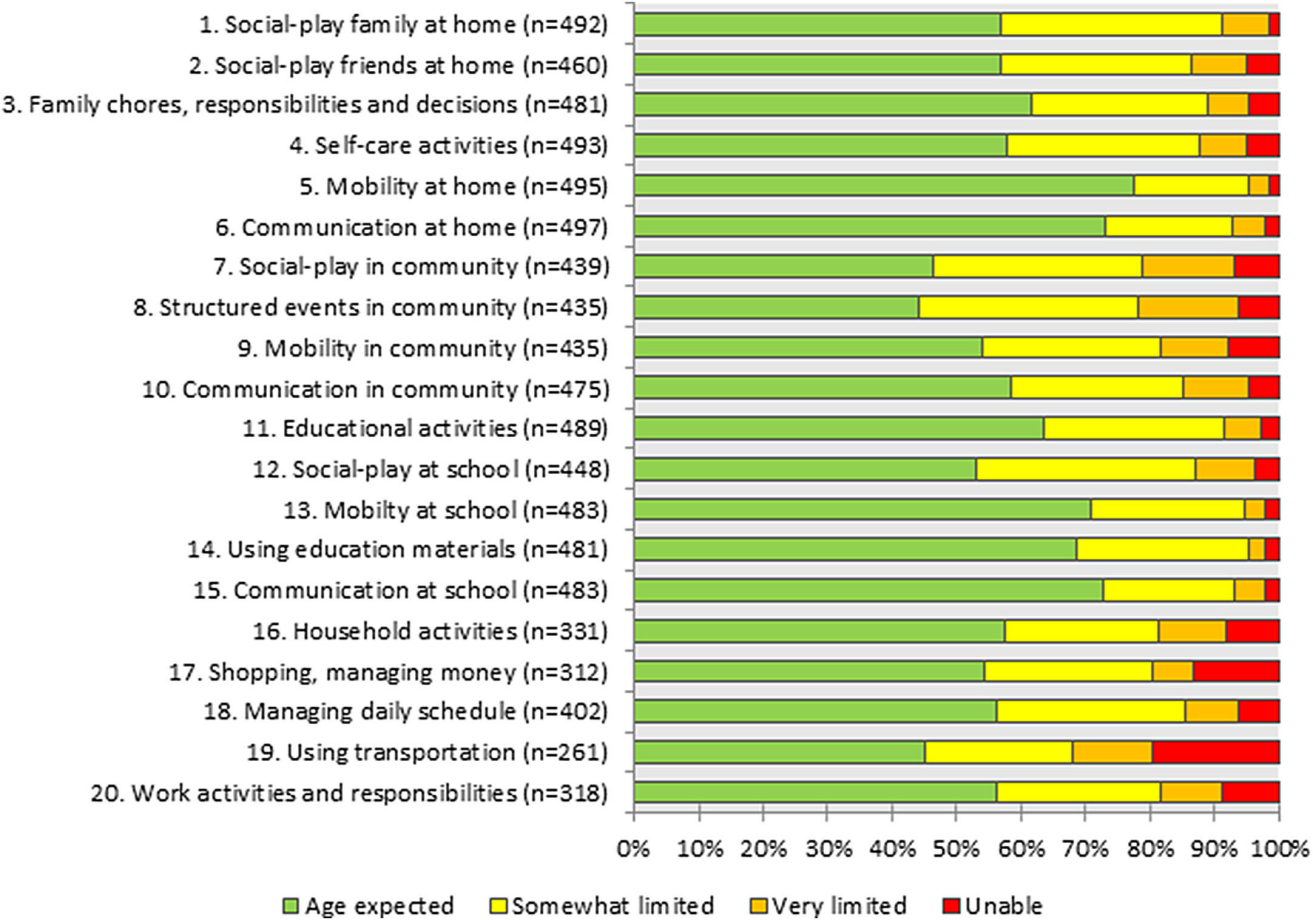
Children's (aged 3–17 years) proxy-reported participation on each of the items of the CASP (*n* = 500).

Figure 2 presents the participation levels of children with VI as reported by their parents on the 20 items of the CASP. Children demonstrated most restrictions in using transportation, structured events in the community, and social, play, and leisure activities with peers. Children were least restricted in mobility at home or school, and communication at home and school.

No significant associations were found for severity of vision loss and any of the subscales of the KIDSCREEN-27, except for better scores for mild VI as compared to no VI on the School Environment subscale as reported by their parents and better scores for severe VI/blind as compared to no VI as reported by the children (Table 4). The latter was only present after correcting for potential confounders. For the CASP, mild VI was significantly associated with better scores in the proxy-report as compared to no VI in the uncorrected model, whereas after correcting for potential confounders, moderate VI and severe VI/blind were significantly associated with worse scores in the proxy-report, as compared to no VI.

**Table 4.** Associations Between Severity of Vision Loss (Mild VI, Moderate VI, or Severe VI/Blind) and the KIDSCREEN-27 and CASP (Sub)Scales as Compared to a Reference Group With no VI

	Uncorrected Model: β (95% CI)			Corrected Model^†^: β (95% CI)		
Dependent Variable^*^	Mild VI^‡^	Moderate VI^‡^	Severe VI/Blind^‡^	Mild VI^‡^	Moderate VI^‡^	Severe VI/Blind^‡^
KIDSCREEN-27 proxy-reported (*n* = 250/246)^§^						
Physical Wellbeing	1.65 (−2.40; 5.69)	1.33 (−2.23; 4.88)	−0.57 (−5.54; 4.41)	0.48 (−3.50; 4.45)	−0.19 (−3.72; 3.34)	0.37 (−4.54; 5.28)
Psychological Wellbeing	1.88 (−2.10; 5.86)	1.98 (−1.52; 5.47)	3.76 (−1.13; 8.65)	1.31 (−2.77; 5.39)	1.26 (−2.36; 4.88)	3.03 (−2.01; 8.08)
Autonomy & Parent Relation	1.29 (−2.06; 4.64)	0.59 (−2.36; 3.53)	0.81 (−3.31; 4.94)	0.69 (−2.62; 9.33)	−0.07 (−3.00; 2.87)	0.18 (−3.90; 4.27)
Social Support & Peers	2.73 (−0.82; 6.27)	1.35 (−1.77; 4.46)	3.99 (−0.37; 8.35)	2.00 (−1.58; 5.59)	0.57 (−2.61; 3.75)	3.89 (−0.55; 8.31)
School Environment	**4.47 (0.75; 8.19)**	0.88 (−2.38; 4.15)	0.94 (−3.64; 5.51)	**3.85 (0.14; 7.56)**	−0.13 (−3.42; 3.17)	0.60 (−3.99; 5.18)
KIDSCREEN-27 self-reported (*n* = 256/245)^§^						
Physical Wellbeing	2.53 (−0.67; 5.72)	0.71 (−2.13; 3.55)	−1.58 (−5.66; 2.50)	2.23 (−1.07; 5.53)	0.39 (−2.58; 3.36)	−0.46 (−4.67; 3.75)
Psychological Wellbeing	−0.44 (−3.59; 2.71)	−0.85 (−3.66; 1.95)	1.21 (−2.82; 5.23)	−1.01 (−4.30; 2.27)	−1.43 (−4.38; 1.52)	0.85 (−3.33; 5.03)
Autonomy & Parent Relation	0.13 (−3.36; 3.61)	−0.44 (−3.54; 2.65)	4.36 (−0.09; 8.80)	−0.16 (−3.79; 3.47)	−0.88 (−4.14; 2.39)	3.71 (−0.92; 8.33)
Social Support & Peers	1.33 (−2.51; 5.18)	−0.30 (−3.72; 3.12)	3.89 (−1.02; 8.80)	0.39 (−3.57; 4.34)	−1.13 (−4.68; 2.42)	3.22 (−1.81; 8.26)
School Environment	−1.09 (−4.52; 2.34)	−0.31 (−3.36; 2.74)	3.75 (−0.63; 8.13)	−1.38 (−4.86; 2.10)	−0.87 (−4.00; 2.27)	**4.81 (0.38; 9.25)**
CASP proxy-reported (*n* = 405/393)^§^	**6.29 (0.68; 11.90)**	1.85 (−3.20; 6.89)	−6.82 (−14.27; 0.63)	0.86 (−3.87; 5.58)	−**4.51 (**−**8.77;** −**0.25)**	−**9.24 (**−**15.48;** −**3.01)**
CASP self-reported (*n* = 71/68)^§^	2.40 (−4.71; 9.52)	1.71 (−4.46; 7.89)	−3.60 (−10.71; 3.52)	2.94 (−4.03; 9.91)	1.88 (−4.38; 8.13)	−1.82 (−9.19; 5.56)

* Higher scores represent better quality of life/participation.

† Corrected for age, gender, comorbidity, and level of education.

‡ Mild VI = logMAR ≤ 0.52, moderate VI = logMAR > 0.52 ≤ 1, severe VI/blindness = logMAR > 1 or visual field ≤10 degrees^29^; and mild VI served as reference.

§ *n* for uncorrected and corrected model, respectively.

Bold face is significant at *P* < 0.05.

## Discussion

This study reports on quality of life and participation of children aged 3 to 17 years with VI, as assessed with the KIDSCREEN-27 and CASP, respectively. Children with VI experienced worse quality of life than population-based samples, which was expressed by worse performance on the subscales Physical Wellbeing and Social Support & Peers, whereas their performance was better on the subscale School Environment. Children with VI also participated to a lesser extent than the population-based samples, but their participation levels were higher than a sample with different chronic conditions or disabilities. Last, this study provides insight into the association between severity of vision loss and quality of life or participation. No differences were found between severity of vision loss and quality of life. However, when compared to no VI, children with more severe levels of VI experienced less participation.

Concerning quality of life, we found mixed effects for the various subscales. Effect sizes were mostly small, although some moderate effect sizes were found, such as on the subscale Physical Wellbeing. The mixed results are in contrast to other studies, in which children with VI often score significantly worse than children with normal vision on (subscales of) quality of life.^20^^–^^22^ We found that children in most age and gender subgroups experienced worse quality of life regarding Physical Wellbeing and Social Support & Peers, confirmed by their parents’ reports. Our findings align with those of other studies, showing that children with VI are less physically active, have more sedentary lifestyles, and poorer physical fitness than children without VI.^4^ Furthermore, studies show that children with VI have fewer friends, perceive the quality of their friendships as lower, have smaller social networks, and more often report feelings of loneliness than sighted counterparts.^6^^,^^39^^–^^41^ A systematic review on interventions in children with VI showed mixed results regarding the effectivity of interventions to improve social skills, but interventions offering physical training or sports camps were effective in increasing physical performance of children with VI.^42^ Implementation of these interventions might increase physical wellbeing of children with VI, whereas more research is warranted into what these interventions add regarding improving social skills and relationships.

There seems to be an age effect for the subscales Psychological Wellbeing and Autonomy & Parent Relation, with younger children experiencing worse quality of life on these subscales than reference groups, whereas older children report a more favorable profile. It is possible that combining these age-groups in our primary analyses leveled out the effects found in the total group of children. Our results are in contrast to the findings of Van Dijk et al., who found that younger children with retinoblastoma experienced a better quality of life than reference populations, whereas older children performed worse.^19^ However, 79% of these children had normal vision.

Both parents and children reported significantly better quality of life for School Environment. However, an age effect seemed present with worse quality of life for the younger children and better quality of life for the older children. Children with VI in the Netherlands often attend regular education where they receive ambulatory counseling. The extra attention these children receive in their education might explain the more favorable experiences of children with VI. The observed age effect might indicate that high schools are better equipped for students with special needs, or that older children are better able to cope with their VI or underestimate the impact of their VI.

Regarding participation, parents reported significantly worse participation of children aged 3 to 11 years than an age-matched population-based sample, and the effect size was large. Compared to a population of children with chronic conditions and disabilities, children aged 12 to 17 years experienced significantly better participation, both reported by children themselves and their parents. Unfortunately, no population-based data for children 12 to 17 years was available. However, the response options of the CASP are formulated in such a way that respondents evaluate their participation while thinking of an age-matched reference population. As such, children without disabilities are expected to report age-expected participation on most items (i.e. resulting in sum scores close to 100).^31^ Considering this, children with VI experience notably worse participation. Over 40% of the children with VI participated worse than age-expected on 13 of the 20 CASP items reported by their parents. A relatively large number of missing responses was observed from parents of younger children on the last five items of the CASP, referring to home and community living activities. The same was observed in a study of De Bock et al., which might indicate that the CASP is less suitable for very young children, or that parents are unable to evaluate young children's participation concerning age expectations.^35^^,^^43^

Our results showed no clear trend for worse quality of life with more severe VI. These findings oppose Chadha and Subramanian, who found a correlation between quality of life and severity of VI in children,^21^ but are in line with research conducted in young adults with VI, which also failed to detect an association between severity of VI and quality of life.^44^ Although misclassification of children in categories of VI might have played a role in the inability to detect a significant association, the KIDSCREEN-27 questionnaire might also not be sensitive enough to the specific problems children with VI encounter. After correcting for potential confounders, worsening participation with more severe VI was observed as reported by parents. However, the percentage of explained variance was low, and therefore other factors (e.g. acceptance and perceived health) might also play a role.

This study has several limitations, including a relatively large group of participants with “No VI” (>35%). One should keep in mind that these children were all registered at low vision services, and thus likely comply to the national guidelines for referral. The categorization of VI was mostly based on visual acuity loss of the better seeing eye, according to definitions of the WHO,^29^ because data on visual field were often lacking or described by subjective phrases, such as “peripheral field loss” or “strong concentrically restricted.” However, classification based on the better seeing eye might not be completely accurate, as interocular differences have an effect on various visual parameters.^45^^–^^47^ Furthermore, in young children, it is often challenging to measure visual acuity and visual field, and diagnoses are not always determined. Moreover, 16% to 17% of the children had cerebral visual impairment (CVI), a term used to cover impaired vision because of brain damage. These children often have normal visual acuity. Thus, some participants might have been misclassified into certain categories of VI, although they were actually having more severe VI. Therefore, the impact of severity of VI on quality of life and participation might be underestimated in our study. Second, the study population was very diverse. Many different causes of VI were reported, including rare genetic disorders and syndromes, and over 40% had some type of comorbidity, which was assessed through an open-ended question. Furthermore, parents differently interpreted the question regarding the time of onset of their child's VI; parents, for instance, stated that time of onset was since birth for a genetic disorder, even if it emerged at a later age. It was therefore not possible to include subgroups of causes of VI and comorbidity in the analyses, although one could argue that the type of ophthalmological condition (e.g. stable or progressive, since birth or later in life) or comorbidity influences quality of life and participation. Last, children completed the questionnaires through face-to-face interviews conducted at their homes, whereas KIDSCREEN-27 reference data were collected through surveys.^30^ Face-to-face interviews are known to cause socially desirable responses,^48^ and indeed children tend to respond more positively on most subscales of the KIDSCREEN-27. However, the use of published reference data can also be considered a strength, as the sample used to collect these data is representative for the population and is much larger than we would be able to collect. Reference data for subscales of the KIDSCREEN-27 was, for example, collected among 1813 to 1862 children and their parents,^30^ which were representative for the Dutch population.

To our knowledge, this is the largest study assessing the quality of life in children with VI and the first to compare participation of children with VI to reference groups. The data were gathered at several vision rehabilitation centers throughout the Netherlands, resulting in a large national cohort. A second strength is the use of psychometrically sound instruments. The KIDSCREEN-27 and CASP have been extensively evaluated.^27^^,^^28^^,^^31^^,^^35^^,^^37^ We re-examined the factor structure of the CASP for this population, indicating a unidimensional scale and replicating the findings of other studies.^31^^,^^35^ Nevertheless, the CASP has originally been developed for children with acquired brain injury, and it remains uncertain whether the content of the CASP is also relevant, comprehensive, and comprehensible for children with VI. Further research is required to investigate whether these key aspects of content validity, which is generally recognized as the most important measurement property of a questionnaire,^49^ are also sufficient in children with VI. Consequently, the results should be interpreted with caution.

In conclusion, this study shows that quality of life of children with VI is affected particularly regarding Physical Wellbeing and Social Support & Peers compared to a reference population. Moreover, compared to a population-based reference group, their participation is considerably worse. After correcting for confounders, more severe vision loss was significantly associated with worse participation, as reported by parents. This study contributes to the understanding of quality of life and participation in children with VI, which is valuable for children themselves, their parents and health care professionals working with these children. Rehabilitation services should focus on those aspects most affected (i.e. physical wellbeing, social life and relationships, participation in community events, and using transportation). Physical wellbeing might be improved by implementing interventions, such as physical training or sports camps, that are likely to be effective. More research into new interventions or changes in existing rehabilitation programs might be warranted to improve social skills and participation of children with VI.

## References

[1] Bourne RRA, Flaxman SR, Braithwaite T, et al. Magnitude, temporal trends, and projections of the global prevalence of blindness and distance and near vision impairment: a systematic review and meta-analysis. *Lancet Glob Health*. 2017; 5: e888–e897. 28779882 10.1016/S2214-109X(17)30293-0

[2] Dale N, Sonksen P. Developmental outcome, including setback, in young children with severe visual impairment. *Dev Med Child Neurol*. 2002; 44: 613–622. 12227616 10.1017/s0012162201002651

[3] Wagner MO, Haibach PS, Lieberman LJ. Gross motor skill performance in children with and without visual impairments - Research to practice. *Res Dev Disabil*. 2013; 34: 3246–3252. 23891733 10.1016/j.ridd.2013.06.030

[4] Augestad LB, Jiang L. Physical activity, physical fitness, and body composition among children and young adults with visual impairments: A systematic review. *Br J Vis Impair*. 2015; 33: 167–182.

[5] Gold D, Shaw A, Wolffe K. The social lives of Canadian youths with visual impairments. *J Visual Impair Blin*. 2010; 104: 431–443.

[6] Kef S. Psychosocial adjustment and the meaning of social support for visually impaired adolescents. *J Visual Impair Blin*. 2002; 96: 22–37.

[7] Tadic V, Hundt GL, Keeley S, Rahi JS, Vision-related Quality of Life Group. Seeing it my way: living with childhood onset visual disability. *Child Care Health Dev*. 2015; 41: 239–248. 24913734 10.1111/cch.12158

[8] Rainey L, Elsman EBM, van Nispen RMA, van Leeuwen LM, van Rens G. Comprehending the impact of low vision on the lives of children and adolescents: a qualitative approach. *Qual Life Res*. 2016; 25: 2633–2643. 27076189 10.1007/s11136-016-1292-8 PMC5010827

[9] Valderas JM, Kotzeva A, Espallargues M, et al. The impact of measuring patient-reported outcomes in clinical practice: a systematic review of the literature. *Qual Life Res*. 2008; 17: 179–193. 18175207 10.1007/s11136-007-9295-0

[10] Chen J, Ou LX, Hollis SJ. A systematic review of the impact of routine collection of patient reported outcome measures on patients, providers and health organisations in an oncologic setting. *BMC Health Serv Res*. 2013; 13: 211. 23758898 10.1186/1472-6963-13-211 PMC3700832

[11] Tijhuis MAR, Picavet HSJ, Hoeymans N. What is quality of life and how is it measured [Wat is kwaliteit van leven en hoe wordt het gemeten]. In: Tijhuis MAR, Picavet HSJ, Hoeymans N, editors. *Public Health Future Forecast [Volksgezondheid Toekomst Verkenning]*. Bilthoven: RIVM; 2002.

[12] Schalock RL, Bonham GS, Verdugo MA. The conceptualization and measurement of quality of life: Implications for program planning and evaluation in the field of intellectual disabilities. *Eval Program Plann*. 2008; 31: 181–190. 18394704 10.1016/j.evalprogplan.2008.02.001

[13] Colver A. Quality of life and participation. *Dev Med Child Neurol*. 2009; 51: 656–659. 19627339 10.1111/j.1469-8749.2009.03321.x

[14] WHO. *The International Classification of Functioning, Disability and Health for Children and Youyh (ICF-CY)*. Geneva, Switzerland: World Health Organization; 2007.

[15] Elsman EBM, van Nispen RMA, van Rens G. Psychometric evaluation of a new proxy-instrument to assess participation in children aged 3-6 years with visual impairment: PAI-CY 3-6. *Ophthalmic Physiol Opt*. 2019; 39: 378–391. 31468574 10.1111/opo.12642 PMC6851879

[16] Tadic V, Cooper A, Cumberland P, Lewando-Hundt G, Rahi JS, Vision-related Quality of Life Group. Measuring the quality of life of visually impaired children: first stage psychometric evaluation of the novel VQoL_CYP instrument. *PLoS One*. 2016; 11: e0146225. 26918329 10.1371/journal.pone.0146225 PMC4768881

[17] Khadka J, Ryan B, Margrain TH, Court H, Woodhouse JM. Development of the 25-item Cardiff Visual Ability Questionnaire for Children (CVAQC). *Br J Ophthalmol*. 2010; 94: 730–735. 20508047 10.1136/bjo.2009.171181

[18] Boulton M, Haines L, Smyth D, Fielder A. Health-related quality of life of children with vision impairment or blindness. *Dev Med Child Neurol*. 2006; 48: 656–661. 16836777 10.1017/S0012162206001381

[19] Van Dijk J, Huisman J, Moll AC, et al. Health-related quality of life of child and adolescent retinoblastoma survivors in the Netherlands. *Health Qual Life Out*. 2007; 5: 65. 10.1186/1477-7525-5-65 PMC2219958 18053178

[20] Wong HB, Machin D, Tan SB, Wong TY, Saw SM. Visual impairment and its impact on health-related quality of life in adolescents. *Am J Ophthalmol*. 2009; 147: 505–511.e1. 19056077 10.1016/j.ajo.2008.09.025

[21] Chadha RK, Subramanian A. The effect of visual impairment on quality of life of children aged 3-16 years. *Br J Ophthalmol*. 2011; 95: 642–645. 20852314 10.1136/bjo.2010.182386

[22] Bathelt J, de Haan M, Dale NJ. Adaptive behaviour and quality of life in school-age children with congenital visual disorders and different levels of visual impairment. *Res Dev Disabil*. 2019; 85: 154–162. 30557846 10.1016/j.ridd.2018.12.003

[23] Chak M, Rahi JS, British Congenital Cataract Interest Group. The health-related quality of life of children with congenital cataract: findings of the British Congenital Cataract Study. *Br J Ophthalmol*. 2007; 91: 922–926. 17244652 10.1136/bjo.2006.109603 PMC1955663

[24] Elsman EB, van Nispen RM, van Rens GH. First stage psychometric testing of a new instrument for adolescents with visual impairment: the Participation and Activity Inventory for Children and Youth (PAI-CY) 13–17 years. *JPRO*. 2020; 4: 1–10. 10.1186/s41687-020-00228-3 PMC7376774 32700170

[25] Elsman EB, Peeters CF, van Nispen RM, van Rens GH. Network analysis of the Participation and Activity Inventory for Children and Youth (PAI-CY) 7–12 years with visual impairment. *Transl Vis Sci Technol*. 2020; 9: 19. 10.1167/tvst.9.6.19 PMC7409088 32821516

[26] Van Rens GHMB, Vreeken HL, Van Nispen RMA. *Guideline visual impairment, rehabilitation and referral [Richtlijn visusstoornissen, revalidatie en verwijzing]*. Nijmegen, the Netherlands: Dutch Society of Ophthalmology [Nederlands Oogheelkundig Gezelschap]; 2011.

[27] de Kloet AJ, Berger MA, Bedell GM, Catsman-Berrevoets CE, van Markus-Doornbosch F, Vliet Vlieland TP. Psychometric evaluation of the Dutch language version of the Child and Family Follow-up Survey. *Dev Neurorehabil*. 2015; 18: 357–364. 24304040 10.3109/17518423.2013.850749

[28] Ravens-Sieberer U, Auquier P, Erhart M, et al. The KIDSCREEN-27 quality of life measure for children and adolescents: psychometric results from a cross-cultural survey in 13 European countries. *Qual Life Res*. 2007; 16: 1347–1356. 17668292 10.1007/s11136-007-9240-2

[29] World Health Organization (WHO). *International Statistical Classification of Diseases and Related Health Problems 10th Revision. Version 2010. Chapter VII, H54: Visual impairment including blindness*. Geneva, Switzerland: World Health Organization; 2010.

[30] Kidscreen. *The KIDSCREEN Questionnaires - Quality of life questionnaires for children and adolescents*. Handbook. Lengerich: KIDSCREEN group; 2006.

[31] Bedell G. Further validation of the Child and Adolescent Scale of Participation (CASP). *Dev Neurorehabil*. 2009; 12: 342–351. 20477563 10.3109/17518420903087277

[32] Rainey L, van Nispen R, van der Zee C, van Rens G. Measurement properties of questionnaires assessing participation in children and adolescents with a disability: a systematic review. *Qual Life Res*. 2014; 23: 2793–2808. 24970678 10.1007/s11136-014-0743-3

[33] Bedell G. *The Child and Adolescent Scale of Participation (CASP): Administration and Scoring Guidelines*. Medford, MA, USA: Tufts University; 2011.

[34] Holbrook A. Acquiescence Response Bias. 2008 2018/01/17. In: *Encyclopedia of Survey Research Methods [Internet]*. Thousand Oaks, California: Sage Publications, Inc. Available from: http://methods.sagepub.com/reference/encyclopedia-of-survey-research-methods.

[35] De Bock F, Bosle C, Graef C, Oepen J, Philippi H, Urschitz MS. Measuring social participation in children with chronic health conditions: validation and reference values of the child and adolescent scale of participation (CASP) in the German context. *BMC Pediatr*. 2019; 19: 125. 31018847 10.1186/s12887-019-1495-6 PMC6482577

[36] Raîche G, Walls TA, Magis D, Riopel M, Blais J-G. Non-graphical solutions for Cattell's scree test. *Methodology*. 2013; 9(1): 23–29.

[37] McDougall J, Bedell G, Wright V. The youth report version of the Child and Adolescent Scale of Participation (CASP): assessment of psychometric properties and comparison with parent report. *Child: Care, Health Dev*. 2013; 39: 512–522. 23763252 10.1111/cch.12050

[38] Cohen J. *Statistical power analysis for the behavioural sciences*. 2nd ed. Hillsdale, New Jersey: Lawrence Earlbaum Associates; 1988.

[39] Salminen AL, Karhula ME. Young persons with visual impairment: Challenges of participation. *Scand J Occup Ther*. 2014; 21: 267–276. 24784723 10.3109/11038128.2014.899622

[40] Koster M, Pijl SJ, Nakken H, Van Houten E. Social participation of students with special needs in regular primary education in the Netherlands. *Int J Disabil Dev Ed*. 2010; 57: 59–75.

[41] Lifshitz H, Hen I, Weisse Z. Self-concept, adjustment to blindness, and quality of friendship among adolescents with visual impairments. *J Visual Impair Blin*. 2007; 101: 96–107.

[42] Elsman EBM, Al Baaj M, van Rens G, et al. Interventions to improve functioning, participation, and quality of life in children with visual impairment: a systematic review. *Surv Ophthalmol*. 2019; 64: 512–557. 30703405 10.1016/j.survophthal.2019.01.010

[43] Bedell GM, Dumas HM. Social participation of children and youth with acquired brain injuries discharged from inpatient rehabilitation: A follow-up study. *Brain Injury*. 2004; 18: 65–82. 14660237 10.1080/0269905031000110517

[44] Elsman EBM, van Rens G, van Nispen RMA. Quality of life and participation of young adults with a visual impairment aged 18-25 years: comparison with population norms. *Acta Ophthalmol*. 2019; 97: 165–172. 30207073 10.1111/aos.13903 PMC6585861

[45] Jennings BJ, Georgeson MA. Adaptation to interocular difference. *J Vision*. 2018; 18: 9. 10.1167/18.5.9 29904784

[46] Chang Y-H, Lee JB, Kim NS, Lee DW, Chang JH, Han S-H. The effects of interocular differences in retinal illuminance on vision and binocularity. *Graefe's Arch Clin Exp Ophthalmol*. 2006; 244: 1083–1088. 16411108 10.1007/s00417-005-0196-z

[47] Baker DH, Wallis SA, Georgeson MA, Meese TS. The effect of interocular phase difference on perceived contrast. *PLoS One*. 2012; 7: e34696. 22485185 10.1371/journal.pone.0034696 PMC3317637

[48] Bowling A. Mode of questionnaire administration can have serious effects on data quality. *J Public Health (Oxf)*. 2005; 27: 281–291. 15870099 10.1093/pubmed/fdi031

[49] Prinsen CA, Mokkink LB, Bouter LM, et al. COSMIN guideline for systematic reviews of patient-reported outcome measures. *Qual Life Res*. 2018; 27: 1147–1157. 29435801 10.1007/s11136-018-1798-3 PMC5891568

